# Protease Activated Receptor-2 Expression and Function in Asthmatic Bronchial Smooth Muscle

**DOI:** 10.1371/journal.pone.0086945

**Published:** 2014-02-13

**Authors:** Benoit Allard, Imane Bara, Guillaume Gilbert, Gabrielle Carvalho, Thomas Trian, Annaig Ozier, Jennifer Gillibert-Duplantier, Olga Ousova, Elise Maurat, Matthieu Thumerel, Jean-François Quignard, Pierre-Olivier Girodet, Roger Marthan, Patrick Berger

**Affiliations:** 1 Univ- Bordeaux, Centre de Recherche Cardio-Thoracique de Bordeaux, U1045, Département de Pharmacologie, Bordeaux, France; 2 INSERM, Centre de Recherche Cardio-Thoracique de Bordeaux, U1045, Bordeaux, France; 3 CHU de Bordeaux, Service d'Exploration Fonctionnelle Respiratoire, Pessac, France; Helmholtz Zentrum München/Ludwig-Maximilians-University Munich, Germany

## Abstract

Asthmatic bronchial smooth muscle (BSM) is characterized by structural remodeling associated with mast cell infiltration displaying features of chronic degranulation. Mast cell-derived tryptase can activate protease activated receptor type-2 (PAR-2) of BSM cells. The aims of the present study were (i) to evaluate the expression of PAR-2 in both asthmatic and non asthmatic BSM cells and, (ii) to analyze the effect of prolonged stimulation of PAR-2 in asthmatic BSM cells on cell signaling and proliferation.

BSM cells were obtained from both 33 control subjects and 22 asthmatic patients. PAR-2 expression was assessed by flow cytometry, western blot and quantitative RT-PCR. Calcium response, transduction pathways and proliferation were evaluated before and following PAR-2 stimulation by SLIGKV-NH_2_ or trypsin for 1 to 3 days.

Asthmatic BSM cells expressed higher basal levels of functional PAR-2 compared to controls in terms of mRNA, protein expression and calcium response. When PAR-2 expression was increased by means of lentivirus in control BSM cells to a level similar to that of asthmatic cells, PAR-2-induced calcium response was then similar in both types of cell. However, repeated PAR-2 stimulations increased the proliferation of asthmatic BSM cells but not that of control BSM cells even following lentiviral over-expression of PAR-2. Such an increased proliferation was related to an increased phosphorylation of ERK in asthmatic BSM cells.

In conclusion, we have demonstrated that asthmatic BSM cells express increased baseline levels of functional PAR-2. This higher basal level of PAR-2 accounts for the increased calcium response to PAR-2 stimulation, whereas the increased proliferation to repeated PAR-2 stimulation is related to increased ERK phosphorylation.

## Introduction

Asthma is a chronic inflammatory disease characterized by bronchial hyperresponsiveness and airway remodeling [1]. Regarding bronchial hyperresponsiveness, the role of a specific increase in the number of mast cells infiltrating the bronchial smooth muscle (BSM) of asthmatics has been put forward [2]. The mechanisms of such mast cell myositis involve a direct chemotactic activity of BSM cells through the production of various cytokines and chemokines, including TGF-β1 [3], CXCL10 [4], and CX_3_CL1 [5]. Moreover, mast cell can adhere to BSM cell by both cell-cell [6] and cell-extra cellular matrix-cell interactions [7]. Features of chronic mast cell degranulation are also present within the asthmatic BSM [8]. Mast cells produce a number of potent mediators, the most abundant of which is the serine protease tryptase (EC 3.4.21.59) [9]. Tryptase has been shown to activate protease activated receptors (PAR) that are expressed at the site of the BSM [10]. Among these receptors, the subtype 2 (PAR-2), plays a major role in bronchial hyperresponsiveness [11], and BSM cell calcium rise [12], [13], as evidenced by pharmacological and RNA interference tools [13]. Taken together, all these findings suggest the presence of an auto-activation loop in asthma, involving mast cells and their mediators including tryptase. Mast cells chronically stimulate PAR-2 in BSM cell inducing bronchial hyperresponsiveness and chemotactic activity, which in turn recruits new mast cells [3].

Regarding airway remodeling, a variety of studies have shown that BSM mass is increased, particularly in severe asthma [14], [15]. Such an increased BSM mass has been associated with a decrease in lung function [15], [16], and related with an increased BSM cell proliferation [15] through a mitochondrial-dependent pathway both *in vitro* and *ex vivo* [14]. As for bronchial hyperresponsiveness, PAR agonists, such as tryptase [17] or YKL-40 [18] have been shown to induce BSM cell proliferation *in vitro* mediated by the subtype PAR-2, as demonstrated by pharmacological and RNA interference tools [17], [18]. However, the potential role of PAR-2 in airway remodeling remains largely unknown in asthma. Indeed, whereas the proliferation of asthmatic BSM cells to a wide range of growth factors, present in fetal calf serum, is increased as compared to that of non asthmatic BSM cells *in vitro* [14], [19], [20], the proliferative response to a single stimulation of PAR-2 by a specific agonist, such as YKL-40, remains unchanged [18]. Moreover, the effect of repeated PAR-2 stimulation on asthmatic BSM cells, a condition that more closely mimics the clinical situation, remains unknown. In addition, in mild to moderate asthmatic bronchi, PAR-2 expression is increased within the epithelium, whereas that in BSM appears unchanged [21]. Nevertheless, the expression of PAR-2 in the BSM remains unknown both in severe asthma *ex vivo* and in all asthmatics *in vitro*.

Therefore, in the present study, both asthmatic and non asthmatic BSM cells were used *in vitro* to evaluate the expression of PAR-2 and the effect of its prolonged stimulation on both calcium and proliferative responses. We found that, asthmatic BSM cells expressed increased baseline levels of functional PAR-2 compared to control BSM cells and that, repeated PAR-2 stimulations increased BSM cell proliferation from asthmatics only, through an ERK-dependent pathway.

## Materials and Methods

### Ethics statement

All patients gave their written informed consent to participate to the study, after the nature of the procedure had been fully explained. The study followed recommendations outlined in the Helsinki Declaration and received the approval from the local ethics committee (“CPP Sud-Ouest et Outre mer IV”).

### Study populations

A total of 22 patients with mild to severe persistent asthma, and 33 non asthmatics were prospectively recruited from the “Centre Hospitalier Universitaire (CHU)” of Bordeaux according to Global Initiative for Asthma criteria [22]. Bronchial specimens were obtained by either fiberoptic bronchoscopy or lobectomy, as previously described [14], [18] (See Table 1 for patients' characteristics).

**Table 1:** Clinical and functional characteristics of subjects.

Characteristics	Non asthmatics	Asthmatics
No. of patients	33	22
Gender (M/F)	26/7	2/20
Age (yr)	61±13	41±17.5
Age range (yr)	19–79	19–72
BMI (kg/m^2^)	26±4.5	26±6.5
Total serum IgE (kUI/L)	ND	473±777
Allergic/Non allergic	0/33	17/5
Exacerbations (nb/yr)	ND	1±1.4
Current treatment		
OCS (n)	0	2
ICS (n)	1	18
Dose of ICS	37±193	1204±877
LABA (n)	0	15
Smoking history		
Never (n)	8	19
Former (n)	10	3
Current (n)	15	0
Pack years	37±28.4	0.7±2.3
FEV_1_		
Liters	3.0±0.6	2.5±0.9
Percentage of predicted value	90.0±14.2	84.3±17.8
FEV_1_∶FVC ratio (% of FVC)	77±6.8	72.7±10.7
FEF_25–75_		
Liters sec^−1^	3±0.9	2.2±1.2
Percentage of predicted value	74±26.8	57.5±25.2

Data are mean ± SD. BMI: body mass index. OCS: oral corticosteroid. ICS: inhaled corticosteroid. LABA: long acting beta2 agonist. FEV1: forced expiratory volume in one second. FVC: forced vital capacity. FEF 25–75: forced expiratory flow between 25 and 75% of FVC.

### Cell culture

Human BSM cells were derived from bronchial specimens, as previously described [14], [18]. Cell purity was assessed by both immunocytochemistry and flow cytometry (Figure S1), on growth arrested cells using serum-free DMEM (PAN Biotech, Brumath, France) for 48 h [18]. All experiments were performed on phenotypically confirmed BSM cells between passages 2 and 5 [14]. For each experiment, BSM cells originating from the same passage were used in both asthmatic and non asthmatic subjects.

A synthetic peptide with a sequence corresponding to the tethered ligand domain of PAR-2 (SLIGKV in humans) has been employed to experimentally activate PAR-2 without proteolytic cleavage [12], [17]. We also used the reverse peptide VKGILS-NH_2_ as negative control for SLIGKV-NH_2_ experiments (PolyPeptide Group, Strasbourg, France). Both SLIGKV-NH_2_ and VKGILS-NH_2_ were used on growth arrested BSM cells at 10^−4^ M and, were changed every 24 h for 1 to 3 days for proliferation, PAR-2 expression, cell transduction and calcium experiments.

PAR-2 expression was assessed by flow cytometry, western blot and quantitative RT-PCR as described previously [13], [18].

### Lentivirus over-expressing PAR-2

The genomic library clone IRATp970H0715D (Source BioScience Lifesciences, Nottingham, United Kingdom), containing PAR-2 ORF cDNA was used to amplify the coding sequence of the gene. The obtained PCR fragment was then cloned into a transfer lentiviral plasmid containing a GFP reporter gene (Plate-forme de vectorologie SFR TransBioMed, Univ Bordeaux, France). As described previously, 3 plasmids were then co-transfected in human embryonic kidney cells (293T) to produce replication-deficient lentiviral particles [18].

### Microspectrofluorimetric measurement of cytosolic calcium

The Ca^2+^-sensitive fluorescent probe indo-1 was used to record changes in intracellular calcium concentration ([Ca^2+^]_i_) in BSM cells, as previously described [12], [13] following stimulation with SLIGKV-NH_2_.

### Cell proliferation

Cell proliferation was evaluated using BrdU incorporation, as previously described [17], [18] following stimulation with either SLIGKV-NH_2_ or VKGILS-NH_2_, for 1 to 3 days.

### Cell transduction

AKT, ERK and p38 phosphorylation were analyzed following 1 to 3 days challenge with SLIGKV-NH_2_, using western blot, as described previously [18], [23].

Mitochondrial mass was assessed by the porin content using western blot, and mitochondrial biogenesis was assessed by peroxysome proliferator-activated receptor gamma co-activator-1 alpha (PGC-1α), nuclear respiratory factor-1 (NRF-1) and mitochondrial transcription factor A (TFAM) contents using both quantitative RT-PCR and western blot, as previously described [14].

### Statistical analysis

The statistical analysis was performed with NCSS software (NCSS 1997®; NCSS Statistical software, Kaysville, Utah). Values are presented as the mean ± SD or SEM. Statistical significance was analyzed by paired Wilcoxon-rank tests and Mann & Whitney tests. A p value<0.05 was considered statistically significant.

## Results

### Asthmatic BSM cells express increased baseline levels of functional PAR-2

**Figure 1:**
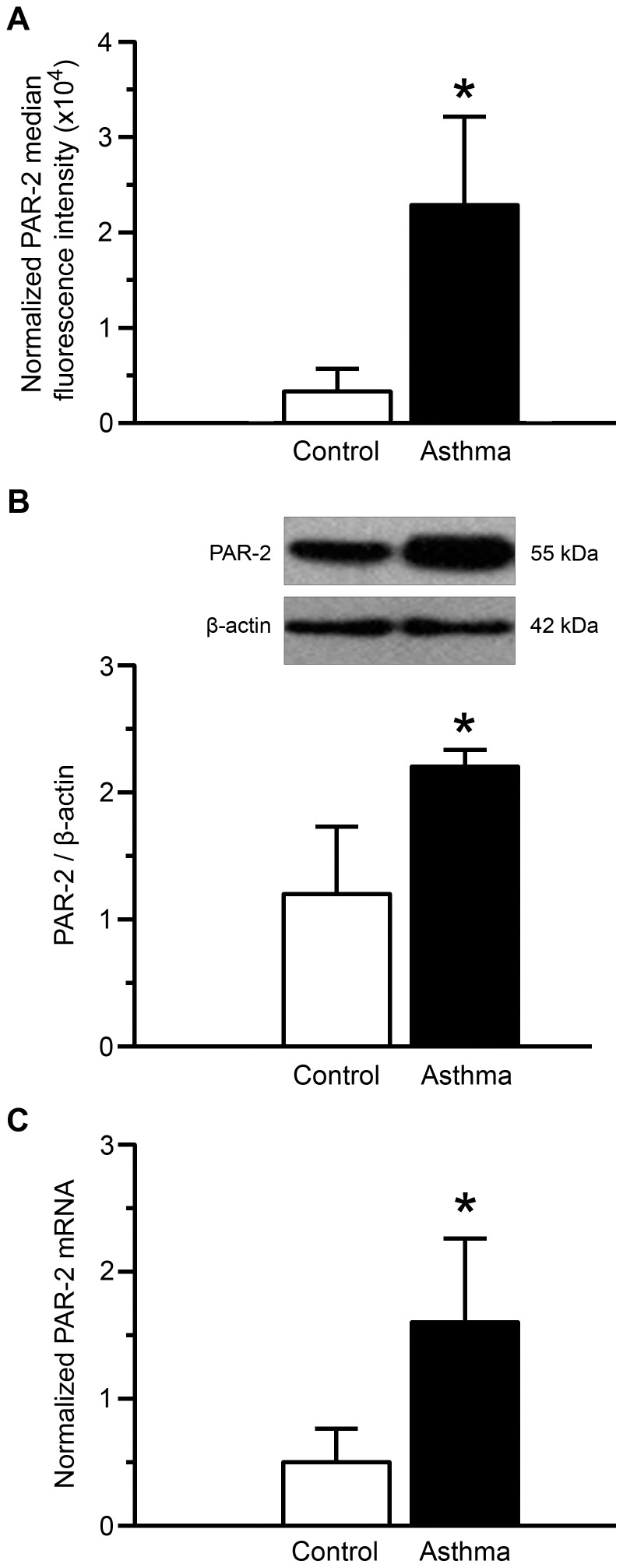
Increased PAR-2 expression in asthmatic bronchial smooth muscle cells. PAR-2 levels were assessed by flow cytometry (A), western blot (B) and quantitative RT-PCR (C). Normalized median fluorescence intensities were calculated by dividing median fluorescence intensity of PAR-2 by that of isotype control (A). Representative blots stained with anti–PAR-2 or anti–β-actin antibodies are shown (B). The RT-PCR expression of PAR-2 was presented as an arbitrary unit and normalized to endogenous references (geometric averaging of three internal control genes; *i.e.* YWHAZ, HPRT-1, and PO) according to GeNorm (C). Bronchial smooth muscle cells were obtained from asthmatic (black bars, n = 7) and control subjects (white bars, n = 7). Results are expressed as mean ± SD. *P<0.05 using Mann & Whitney test.

PAR-2 expression was measured in BSM cells at both protein and mRNA levels. Using flow cytometry, PAR-2 surface expression was increased in asthmatic BSM cells compared to controls (Mean fluorescence intensity: 2.3×10^4^±0.9 vs. 0.3×10^4^±0.2, respectively) (Figure 1A). Moreover, the total PAR-2 protein and mRNA expressions were also increased in asthmatic BSM cells compared to controls using western blot (Figure 1B) and quantitative RT-PCR (Figure 1C), respectively. Similar findings were observed in growth arrested BSM cells, as well as, in BSM cells from passages 2 to 5 (data not shown).

**Figure 2:**
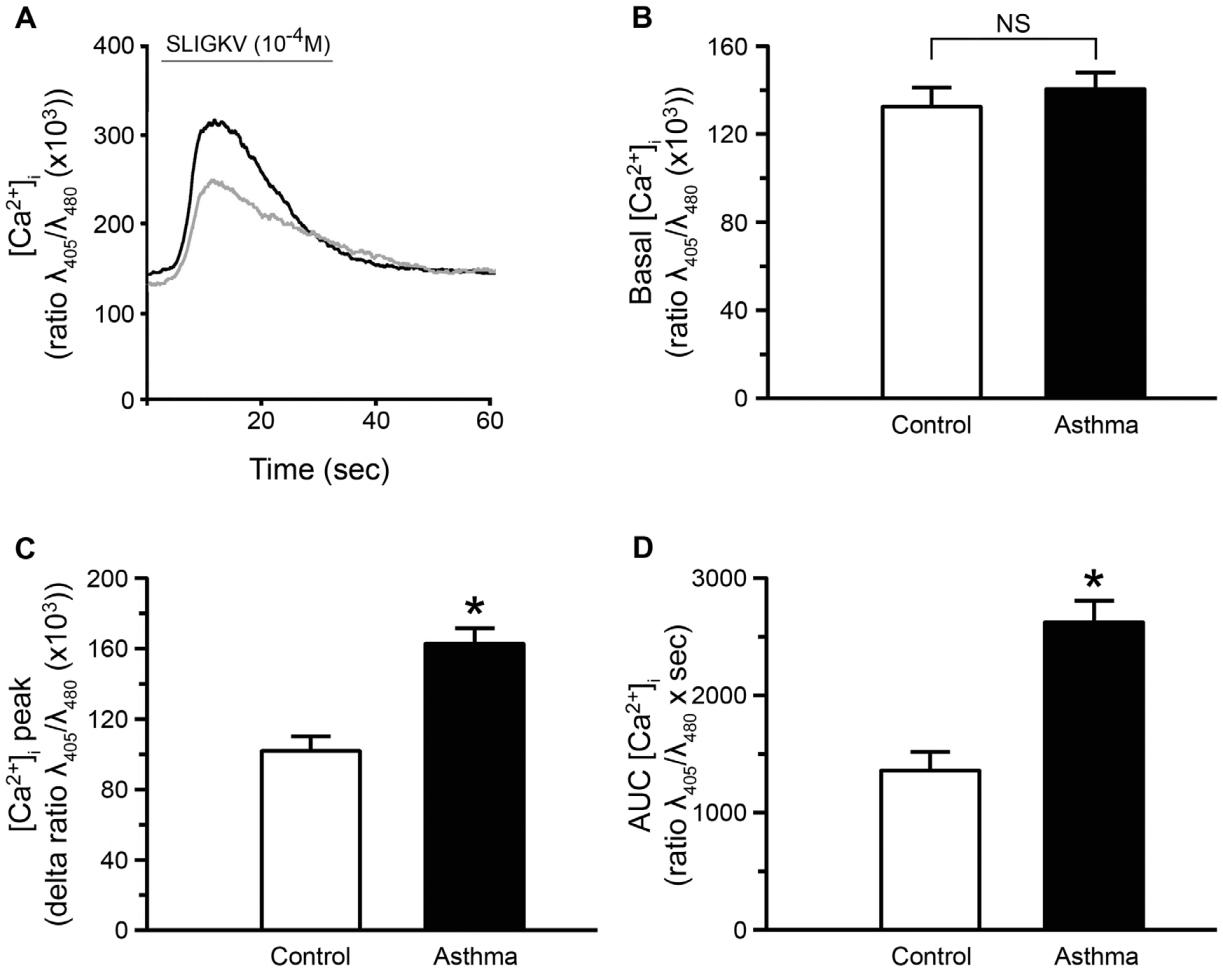
Increased PAR-2 dependent calcium response in asthmatic bronchial smooth muscle cells. Representative intracellular calcium responses following stimulation by 10^−4^ M SLIGKV-NH_2_ for 30 sec are presented in bronchial smooth muscle cells from asthmatic (black line) or control subjects (grey line) (A). Basal calcium concentration (Basal [Ca^2+^]_i_, B), relative calcium response ([Ca^2+^]_i_ peak, C) and area under the curve (AUC [Ca^2+^]_i_, D) were assessed from cell response to 10^−4^ M SLIGKV-NH_2_. Bronchial smooth muscle cells were obtained from asthmatic (black bars, n = 3) and control subjects (white bars, n = 3). Results are expressed as mean ± SEM from a range of 14 to 41 cells per patient. *P<0.05 using Mann & Whitney test.

To determine whether PAR-2 was functional in control and asthmatic BSM cells, we measured PAR-2 induced [Ca^2+^]_i_ rise in growth arrested cells following SLIGKV-NH_2_ (Figure 2A). Basal [Ca^2+^]_i_ was similar in asthmatic and control BSM cells (Figure 2B). We checked that the calcium concentration was stable for at least 60 sec, under baseline condition (Figure S2A). The calcium variation, under this unstimulated condition, was also similar in asthmatic and control BSM cells (Figure S2B). However, calcium peak induced by the PAR-2 agonist peptide SLIGKV-NH_2_ was significantly increased in asthmatic BSM cells compared to controls (160±8 vs. 100±8 delta ratio λ_405_/λ_480_ (×1000), respectively) (Figure 2C). Similarly, the area under the calcium curves was also significantly greater in asthmatic than in control cells (Figure 2D). Moreover, the activation of PAR-2 by trypsin induced similar results (Figure S3). Conversely, the effect of the reverse peptide VKGILS-NH_2_, which does not activate PAR-2, was unchanged in asthmatic compared to control cells (Figure S2C).

### PAR-2 lentiviral over expression enhances PAR-2 calcium response in control BSM cells

**Figure 3:**
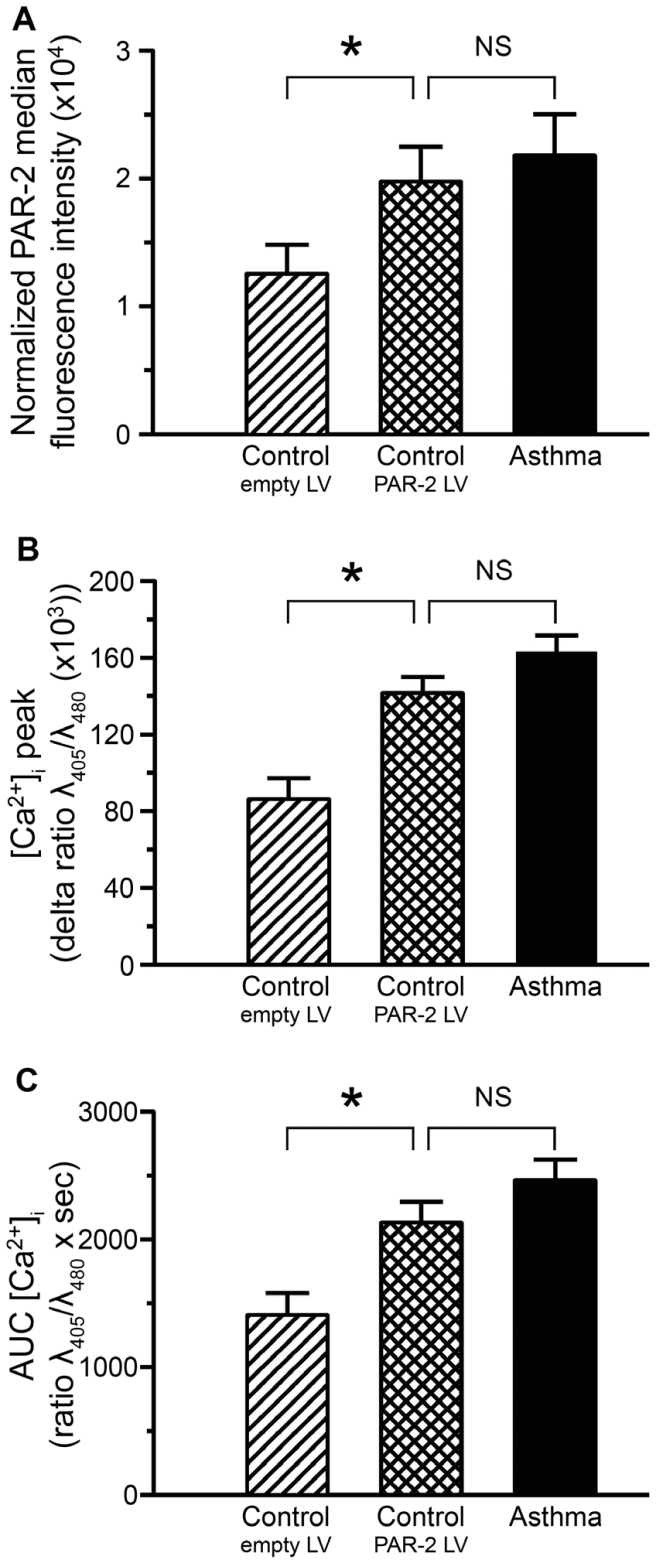
Increased PAR-2 dependent calcium responses in control bronchial smooth muscle cells over-expressing PAR-2. The effects of lentivirus over-expressing PAR-2 (squared bars) in control bronchial smooth muscle cells were evaluated as compared to both control bronchial smooth muscle cells transduced by control lentivirus (hatched bars) and non transduced asthmatic bronchial smooth muscle cells (black bars). PAR-2 surface protein expression was assessed by flow cytometry (A). Relative calcium response ([Ca^2+^]_i_ peak, B) and area under the curve (AUC [Ca^2+^]_i_, C) were assessed by microspectrofluorimetry from the cell response to 10^−4^ M SLIGKV-NH_2_. Bronchial smooth muscle cells were obtained from asthmatic (black bars, n = 3) and control subjects (white bars, n = 3). Results are expressed as mean ± SEM. Calcium responses were obtained from a range of 9 to 35 cells per patient. * *P*<0.05 using paired Wilcoxon-rank tests and Mann & Whitney test.

To confirm the role of higher basal levels of PAR-2 in the increased calcium response observed in asthmatic BSM cells, we used a lentiviral strategy designed to enhance PAR-2 expression in control cells. We first verified lentiviral efficacy using quantitative RT-PCR and flow cytometry. Transduction of control BSM cells with lentiviral PAR-2 particles dramatically increased the transcription of PAR-2 (data not shown). The expression of lentiviral particles was additionally assessed by the presence of GFP in more than 82% of transduced BSM cells (data not shown). Quantitatively, using flow cytometry, we verified that PAR-2 protein expression was increased in these transduced control BSM cells to a level similar to that of asthmatic cells (Figure 3A). We then assessed the functional consequences of this lentiviral PAR-2 over-expression on calcium response. SLIGKV-induced [Ca^2+^]_i_ response was significantly enhanced in control BSM cells with lentiviral PAR-2 over-expression compared to control BSM cells transduced with control lentiviral particles (Figure 3B & C). The observed calcium response was similar to that of asthmatic BSM cells, which expressed increased baseline levels of PAR-2 (Figure 3B & C). Moreover, the pattern of PAR-2-induced calcium response upon activation by trypsin was similar to that observed upon activation by SLIGKV-NH_2_ (Figure S3). As previously observed, the effect of the reverse peptide VKGILS-NH_2_ remained unchanged in control BSM cells with lentiviral PAR-2 over-expression compared to control BSM cells transduced with control lentiviral particles (data not shown) and similar to that under unstimulated condition (Figure S2B).

### Asthmatic BSM cell proliferation is enhanced following PAR-2 stimulation through an ERK-dependent pathway

**Figure 4:**
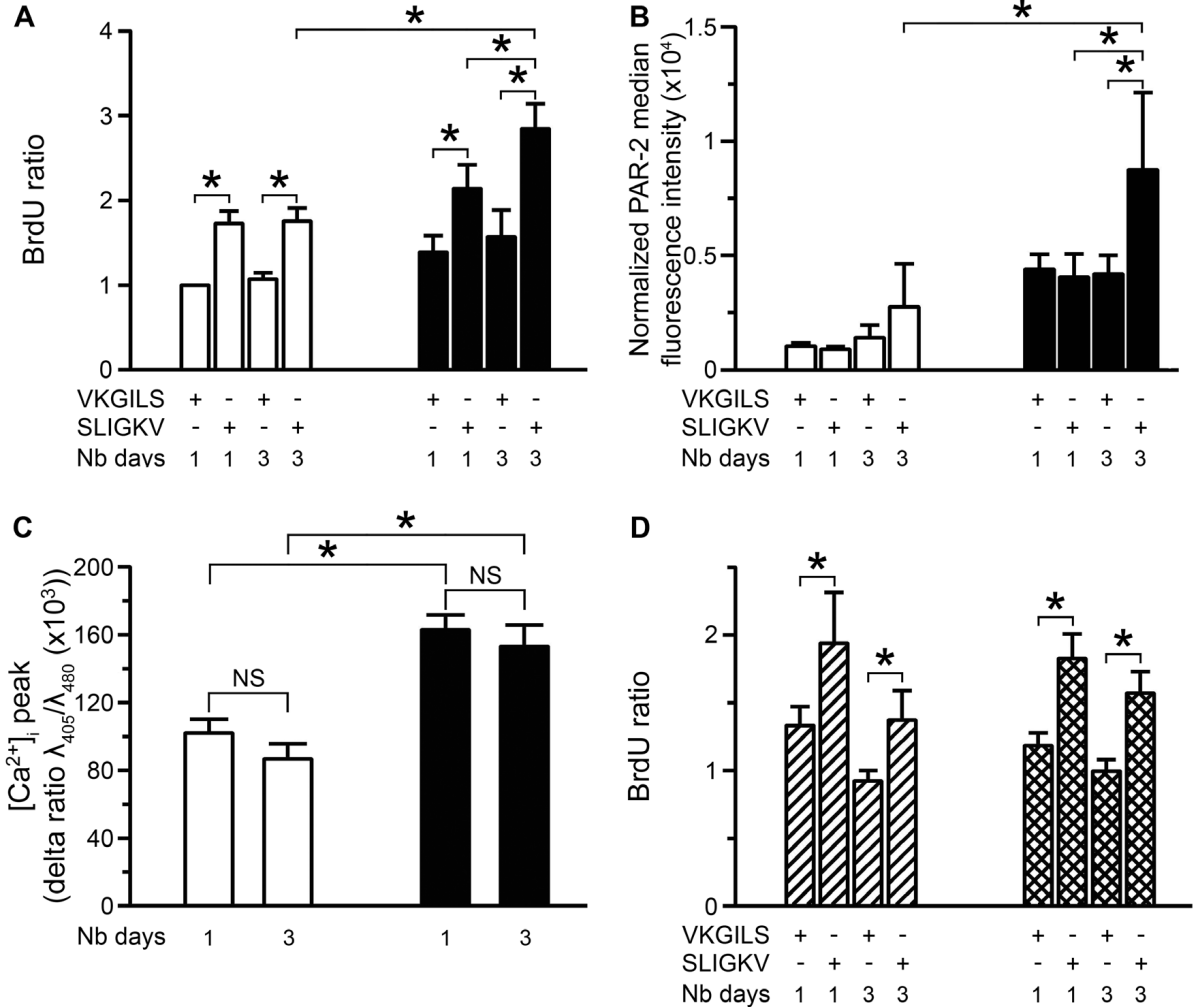
Increased asthmatic bronchial smooth muscle cell proliferation following repeated PAR-2 stimulations. Proliferation was measured using BrdU incorporation following stimulation for 1 or 3 days by 10^−4^ M SLIGKV-NH_2_ or VKGILS-NH_2_ (A). Bronchial smooth muscle cells were obtained from asthmatic (black bars, n = 5) and control subjects (white bars, n = 5). PAR-2 expression was assessed by flow cytometry following stimulation for 1 or 3 days by 10^−4^ M SLIGKV-NH_2_ or VKGILS-NH_2_ (B). Normalized median fluorescence intensities were calculated by dividing median fluorescence intensity of PAR-2 by that of isotype control. Bronchial smooth muscle cells were obtained from asthmatic (black bars, n = 6) and control subjects (white bars, n = 6). Relative calcium response was assessed by microspectrofluorimetry from the cell response to 10^−4^ M SLIGKV-NH_2_ following stimulation for 1 or 3 days by 10^−4^ M SLIGKV-NH_2_ (C). Bronchial smooth muscle cells were obtained from asthmatic (black bars, n = 3) and control subjects (white bars, n = 3). Calcium responses were obtained from a range of 16 to 35 cells per patient. Proliferation was measured using BrdU incorporation following stimulation for 1 or 3 days by 10^−4^ M SLIGKV-NH_2_ or VKGILS-NH_2_ (D). Bronchial smooth muscle cells obtained from control subjects were transduced with control lentivirus (hatched bars, n = 6) or lentivirus over-expressing PAR-2 (squared bars, n = 6). Results are expressed as mean ± SEM. *P<0.05 using paired Wilcoxon-rank tests and Mann & Whitney tests.

We evaluated the proliferation of asthmatic and control BSM cells upon PAR-2 stimulation. Proliferation of asthmatic BSM cells was significantly enhanced following 3 days of stimulation with SLIGKV-NH_2_ (Figure 4A) or trypsin (Figure S4) compared to both control cells and asthmatic BSM cells following 1 day of stimulation. Moreover, PAR-2 expression was increased in asthmatic BSM cells following 3 days of stimulation with SLIGKV-NH_2_ compared to both control cells and asthmatic BSM cells following 1 day of stimulation (Figure 4B). In contrast, PAR-2-dependent calcium response, following 3 days of stimulation with SLIGKV-NH_2_ or trypsin, remained similar in terms of both calcium peak (Figure 4C or Figure S5) and area under the curve (data not shown) to that following 1 day of stimulation in both types of BSM. In addition, SLIGKV-NH_2_ or trypsin failed to increase proliferation in control BSM cells over-expressing PAR-2 (Figure 4D or Figure S4), suggesting that additional pathways are specifically involved in asthmatic BSM cells that account for the increased proliferation. Indeed, the EC50 of SLIGKV-NH_2_ for cell proliferation was similar in control BSM cells i.e. 2.7×10^−6^ M (1.2; 6.0), in control BSM cells over-expressing PAR-2 i.e. 3.6×10^−6^ M (0.5; 23.8) and in asthmatic BSM cells i.e. 1.7×10^−6^ M (0.2; 11.4) (with values presented as means (n = 5 controls and n = 6 asthmatics) with 95% confidence limits in parentheses, p>0.05 using Wilcoxon rank test and Mann & Whitney test). Along the same line, lentiviral PAR-2 over-expression in control BSM cells did not increase either mitochondrial mass (Figure S6A) or mitochondrial biogenesis assessed by the levels of TFAM at both protein (Figure S6B) and mRNA levels (Figure S6C). Moreover, the level of the upstream transcription factors (*i.e.*, NRF-1 and PGC-1α) was not altered in this experimental condition (data not shown).

**Figure 5:**
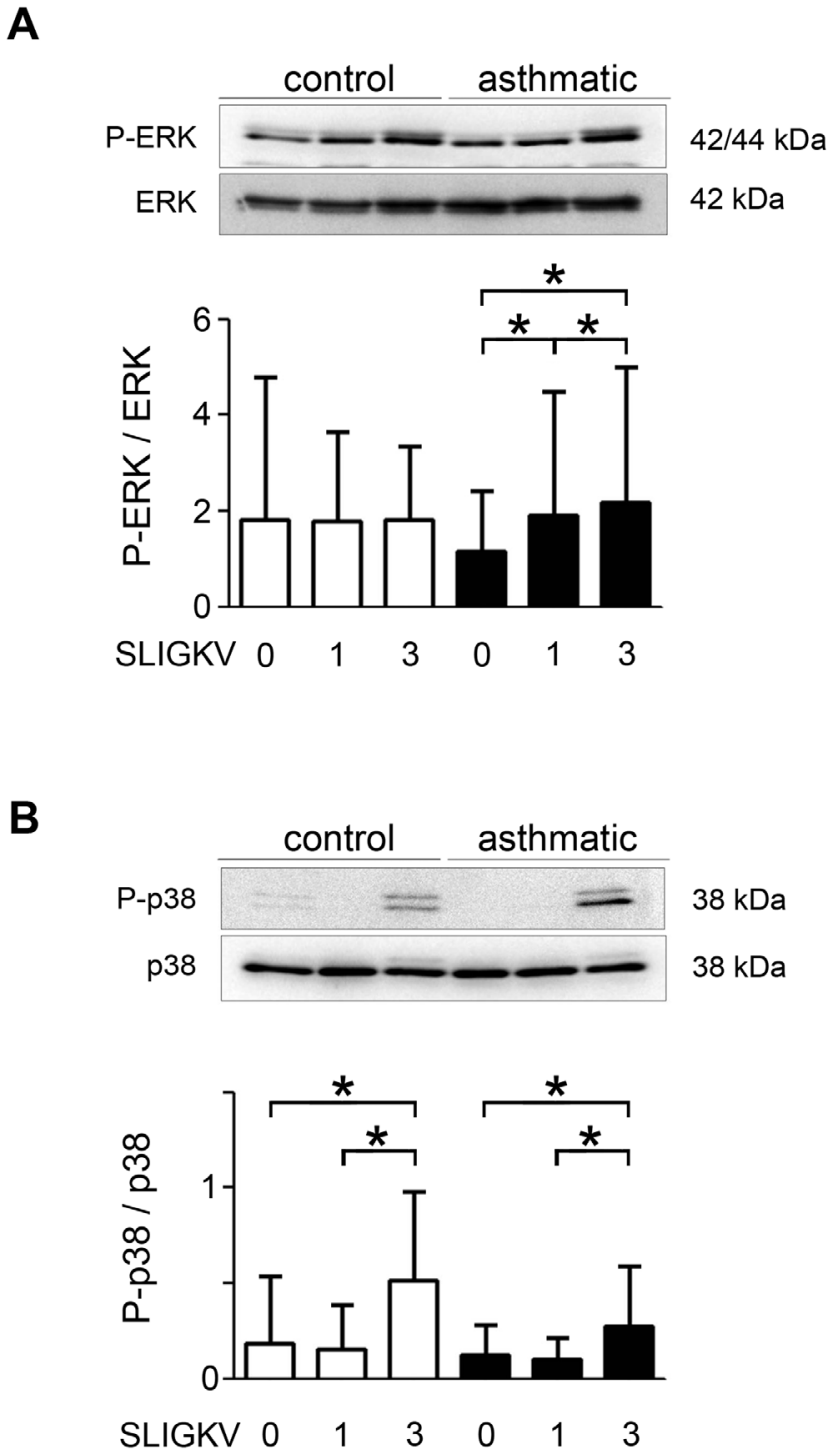
Increased asthmatic bronchial smooth muscle cell phosphorylation of ERK and p38 following repeated PAR-2 stimulations. Phosphorylation of ERK (A) and p38 (B) was measured using western blot following stimulation for 0, 1 or 3 days by 10^−4^ M SLIGKV-NH_2_. Representative blots stained with anti-Phospho-ERK (P-ERK), anti–ERK, anti-Phospho-p38 (P-p38) and anti-p38 antibodies are shown. Bronchial smooth muscle cells were obtained from asthmatic (black bars, n = 8) and control subjects (white bars, n = 7). Results are expressed as mean ± SD. *P<0.05 using paired Wilcoxon-rank tests.

We then evaluated the phosphorylation of various PAR-2-dependent transductions pathways (*i.e.* ERK, p38 and AKT) using western blot. Phosphorylation of ERK was significantly increased in asthmatic BSM cells following 3 days of stimulation with SLIGKV-NH_2_ compared to asthmatic BSM cells either un-stimulated or stimulated for 1 day (Figure 5A and Figure S7A). The role of ERK phosphorylation was confirmed by the significant effect of ERK inhibitor PD98059 (Figure S8). Conversely, 3 days of stimulation of control BSM cells did not alter ERK phosphorylation. Regarding the role of p38, on the one hand, its phosphorylation was significantly increased in both asthmatic and non asthmatic BSM cells following 3 days of stimulation with SLIGKV-NH_2_ (Figure 5B and Figure S7B), but, on the other hand, the p38 inhibitor SB203580 was unable to decrease PAR-2 dependent BSM cell proliferation (Figure S8). Finally, the phosphorylation of AKT was unchanged in both asthmatic and control BSM cells (data not shown).

## Discussion

In this study, we have demonstrated, for the first time that asthmatic BSM cells express increased baseline levels of functional PAR-2. These higher basal levels of PAR-2 account for the increased calcium response to PAR-2 stimulation, whereas the increased proliferation to repeated PAR-2 stimulations is related to increased ERK phosphorylation.

Regarding the level of PAR-2 in asthmatic BSM cells, this is the first study demonstrating an increased expression *in vitro*. A previous study performed *ex vivo* in mild to moderate asthmatics reported an increased PAR-2 expression within the epithelium but not in BSM [21]. In this study, patients with various asthma severities have been included and primary cultured BSM cells from these patients consistently exhibited higher basal levels of PAR-2. However, it should be noted that non asthmatics appeared to be older with higher male to female ratio and higher smoker to never smoker ratio as compared to asthmatics. These differences could be confounding factors in our study. These increased baseline levels of PAR-2 have been measured at both protein and mRNA levels. We did not find any significant correlation between PAR-2 expression and asthma severity. However, the study has not been enough powered to perform such correlation analysis. We paid a special attention to avoid cell culture artifacts. Indeed, similar PAR-2 expression was found in BSM cells from various passages in both FCS and serum deprived culture medium. However, repeated PAR-2 stimulations with SLIGKV-NH_2_ for 3 days was able to further increase PAR-2 expression only within asthmatic BSM cells. Nevertheless, an additional study is required to completely avoid cell culture artifacts. Such a study should evaluate the BSM PAR-2 expression *ex vivo* in a large cohort of asthmatic patients with various severities.

Several studies have shown that asthmatic BSM cell proliferation is increased in response to FCS [14], [19], [20]. However, when looking at the proliferative response to specific agonists such as YKL-40 or PDGF, we previously demonstrated that the proliferation is similar in asthmatic and non asthmatic BSM cells [18]. In the present study, as in that previously published [18], we observed that the proliferation induced by a single stimulation with the PAR-2 agonist peptide SLIGKV-NH_2_ is similar in asthmatic and non asthmatic BSM cells. To further analyze this discrepancy, we have examined the effect of repeated stimulations with SLIGKV-NH_2_ for 3 days and observed that, under such conditions, the proliferation was increased only in asthmatic BSM cells. This increased BSM cell proliferation has been associated with a concomitant increased PAR-2 expression within asthmatic BSM cells. Since it has been previously demonstrated that PAR-2 desensitized quickly in BSM cells [12], [24], in the present study, the agonist peptide SLIGKV-NH_2_ was renewed every 24 h, which is largely enough to allow SLIGKV-NH_2_ to be degraded in the culture medium and new PAR-2 to be synthesized and localized at the plasma membrane of BSM cells [25], [26]. As a consequence, calcium responses, induced by a short stimulation with SLIGKV-NH_2_, were similar in BSM cells after 1 day or 3 days of chronic stimulation with the same agonist peptide, suggesting that desensitization did not occur under these experimental conditions. We do believe that repeated *in vitro* stimulations more likely correspond to the *in vivo* chronic PAR-2 stimulation. Indeed, we have previously observed *ex vivo* typical features of mast cell degranulation within the asthmatic BSM layer [8].

We have previously demonstrated that, in severe asthmatic BSM, the increased FCS-induced BSM cell proliferation is related to an altered calcium signaling through an abnormal extra-cellular calcium entry [14]. Subsequently, Mahn *et al.* demonstrated that, the expression of the sarco/endoplasmic reticulum calcium pump (*i.e.*, SERCA2) is decreased in the BSM from moderate asthmatics [27]. Moreover, knocking down SERCA2 increased non asthmatic BSM cell proliferation [27]. These two studies strongly argue in favor of a direct relationship between calcium response and BSM cell proliferation induced by FCS. In the present study, both PAR-2-dependent calcium response and cell proliferation were increased in asthmatic BSM cells. However, whereas PAR-2 over-expression reproduced the increased calcium response to SLIGKV-NH_2_ in non asthmatic BSM cells, it failed to increase BSM cell proliferation. These results suggest that, the sole over-expression of a receptor coupled to calcium signaling is not enough to increase BSM cell proliferation. This lack of proliferative effect of PAR-2 over-expression was not due to a difference in either the EC50 of SLIGKV-NH_2_ or the level of PAR-2 expression. Indeed, we paid a special attention to optimize the experimental conditions, in order to obtain a similar PAR-2 expression level in non asthmatic BSM cells following lentiviral transduction to that in asthmatic BSM cells. Alternatively, one may suggest that, this lack of proliferative effect could be related to a non specific effect of lentivirus following transduction. This is unlikely since, our data were always compared to those obtained in response to a control lentivirus only lacking PAR-2 cDNA. In addition, this control lentivirus altered neither the calcium response nor the proliferation rate compared to non infected BSM cells. Moreover, since lentiviruses induce a stable protein expression, experiments were performed long time after the stress of transduction (*i.e.*, 7 days later).

Since PAR-2 dependent increased asthmatic BSM cell proliferation was not reproduced by lentiviral PAR-2 over-expression in control BSM cells despite the similar effect on calcium signaling, we also examined PAR-2 downstream signaling transduction pathways. The phosphorylation of ERK appears to be involved since the increased amount of phospho-ERK was restricted to asthmatic BSM cells and, its inhibition by PD98059 inhibited PAR-2 dependent asthmatic BSM cell proliferation. In contrast, the role of p38 could not be demonstrated since, increased amount of phospho-p38 was observed in both asthmatic and non asthmatic BSM cells and, its inhibition by SB203580 did not decrease PAR-2 dependent asthmatic BSM cell proliferation. The current results also point out a striking difference between, asthmatic BSM cells with higher basal levels of PAR-2 and, lentiviral-induced PAR-2 over-expression in non asthmatic BSM cells. Indeed, we have previously demonstrated that, in severe asthmatic BSM, the increased FCS-induced cell proliferation is related to an increased mitochondrial biogenesis [14], which was not found in non asthmatic BSM cells with lentiviral-induced PAR-2 over-expression.

In conclusion, asthmatic BSM cells are characterized by an increased expression of functional PAR-2, the prolonged stimulation of which may contribute to many pathophysiological patterns present in asthma such as bronchial hyperresponsiveness and BSM remodeling. However, the higher basal levels of PAR-2 account for the increased calcium response but are not sufficient to explain increased BSM cell proliferation, which needs the additional up-regulation of ERK phosphorylation. Blocking BSM PAR-2 and/or its transduction pathways could be interesting and promising targets for therapeutic intervention in asthma.

## Supporting Information

Figure S1 Bronchial smooth muscle cell phenotyping. Bronchial smooth muscle phenotype was assessed using both immunocytochemistry (A–F) and flow cytometry (G–H). Representative confocal microscopic images of cultured cells after 3-dimensional reconstruction of 20 sections of 0.25 microns thick (original magnification, ×600; scale bars = 20 µm). Cells were obtained from a control subject (A, B and C) or an asthmatic patient (D, E and F). Cells were stained with anti-cytokeratin 18 (A and D), anti-α-smooth muscle actin (α-SM-actin, B and E) or anti-calponin (C and F) primary antibodies, and by appropriate secondary antibodies (Alexa Fluor 488). Nuclei were stained in blue with DAPI. Representative flow cytometry histograms were obtained using bronchial smooth muscle cells from a control subject. α-smooth muscle-actin (G) or calponin (H) expression was assessed using irrelevant antibodies (gray lines) or specific antibodies (green lines).
(TIF)

Figure S2 Calcium responses under baseline and VKGILS stimulations. Representative intracellular calcium baselines are presented in bronchial smooth muscle cells from asthmatic (black line) or control subject (grey line) (A). Relative calcium response ([Ca^2+^]_i_ variations were assessed under baseline condition (B). The effects of lentivirus over-expressing PAR-2 (squared bars, n = 4) in control bronchial smooth muscle cells were evaluated as compared to both control bronchial smooth muscle cells transduced by control lentivirus (hatched bars, n = 4), control bronchial smooth muscle cells non transduced (white bars, n = 4) and asthmatic bronchial smooth muscle cells (black bars, n = 4). Results are expressed as mean ± SEM from a range of 12 to 19 cells per patient. Representative intracellular calcium responses following stimulation by 10^−4^ M VKGILS-NH_2_ for 30 sec are presented in bronchial smooth muscle cells from asthmatic (black line) or control subjects (grey line) (C). NS *P* non significant using Mann & Whitney test.
(TIF)

Figure S3 Increased trypsin-related calcium response in asthmatic bronchial smooth muscle cells. Representative intracellular calcium responses following stimulation by 3 U/ml trypsin for 30 sec are presented in asthmatic bronchial smooth muscle cells (bold black line), control bronchial smooth muscle cells (grey line) or control bronchial smooth muscle cells transduced with PAR-2 lentivirus (black line) (A). Basal calcium concentration (Basal [Ca^2+^]_i_, B), relative calcium response ([Ca^2+^]_i_ peak, C) and area under the curve (AUC [Ca^2+^]_i_, D) were assessed from cell response to 3 U/ml trypsin. Non transduced bronchial smooth muscle cells were obtained from asthmatic (black bars, n = 4) and control subjects (white bars, n = 4). PAR-2 lentivirus-transduced bronchial smooth muscle cells were obtained from control subjects (squared bars, n = 4). Results are expressed as mean ± SEM from a range of 22 to 46 cells per patient. *P<0.05 using Mann & Whitney test.
(TIF)

Figure S4 Increased asthmatic bronchial smooth muscle cell proliferation following repeated PAR-2 stimulations with trypsin. Proliferation was measured using BrdU incorporation following stimulation for 1 or 3 days by 30 mU/ml trypsin. Bronchial smooth muscle cells were obtained from asthmatic (black bars, n = 3) and control subjects (white bars, n = 3). Bronchial smooth muscle cells obtained from control subjects were also transduced with lentivirus over-expressing PAR-2 (squared bars, n = 3). Results are expressed as mean ± SEM. *P<0.05 using paired Wilcoxon-rank tests or Mann & Whitney tests.
(TIF)

Figure S5 Increased trypsin-related calcium response in asthmatic bronchial smooth muscle cells under repeated stimulations. Relative calcium response ([Ca^2+^]_i_ peak) were assessed by microspectrofluorimetry from the cell response to 3 U/ml trypsin after either 1 day stimulation or 3 days stimulation with 30 mU/ml trypsin. Bronchial smooth muscle cells were obtained from asthmatic (black bars, n = 4) and control subjects (white bars, n = 4). Results are expressed as mean ± SEM. Calcium responses were obtained from a range of 20 to 40 cells per patient. * *P*<0.05 using Mann & Whitney tests. NS non significant using paired Wilcoxon-rank tests.
(TIF)

Figure S6 Unaltered mitochondrial biogenesis in control bronchial smooth muscle cells over-expressing PAR-2. The effects of lentivirus over expressing PAR-2 (squared bars) in control bronchial smooth muscle cells were compared to control bronchial smooth muscle cells transduced by control lentivirus (hatched bars). Porin level was assessed by western blot (A) and mitochondrial transcription factor A (TFAM) levels were assessed by both western blot (B) and quantitative RT-PCR (C). Representative blots stained with anti–porin, anti-TFAM or anti–β-actin antibodies are shown (A and B). Bronchial smooth muscle cells were obtained from control subjects (white bars, n = 4 for A, B, n = 6 for C). Results are expressed as mean ± SEM. *P<0.05 using paired Wilcoxon-rank tests.
(TIF)

Figure S7 Increased asthmatic bronchial smooth muscle cell phosphorylation of ERK and p38 following repeated PAR-2 stimulations. Phosphorylation of ERK (A) and p38 (B) was measured using western blot following stimulation for 0, 1 or 3 days by 10^−4^ M SLIGKV-NH_2_. Representative blots stained with anti-Phospho-ERK (P-ERK), anti-Phospho-p38 (P-p38) and anti–β-actin antibodies are shown. Bronchial smooth muscle cells were obtained from asthmatic (black bars, n = 9) and control subjects (white bars, n = 5). Results are expressed as mean ± SD. *P<0.05 using paired Wilcoxon-rank tests.
(TIF)

Figure S8 ERK inhibition decreased asthmatic bronchial smooth muscle cell proliferation following repeated PAR-2 stimulations. Cell proliferation was measured using BrdU incorporation following stimulation for 3 days by 10^−4^ M SLIGKV-NH_2_ in the absence or in the presence of ERK inhibitor (PD 98059) or p38 inhibitor (SB 203580). Bronchial smooth muscle cells were obtained from asthmatic patients (black bars, n = 4). Results are expressed as mean ± SEM of percentage of cell proliferation in the absence of inhibitor. *P<0.05 using paired Wilcoxon-rank tests.
(TIF)

Methods S1 Supporting materials and methods.
(DOC)
