# Supplementary figures and images for: Protease Activated Receptor-2 Expression and Function in Asthmatic Bronchial Smooth Muscle
Source: PLoS One. 2014 Feb 13;9(2):e86945. doi: 10.1371/journal.pone.0086945 (PMC3923726; doi:10.1371/journal.pone.0086945)

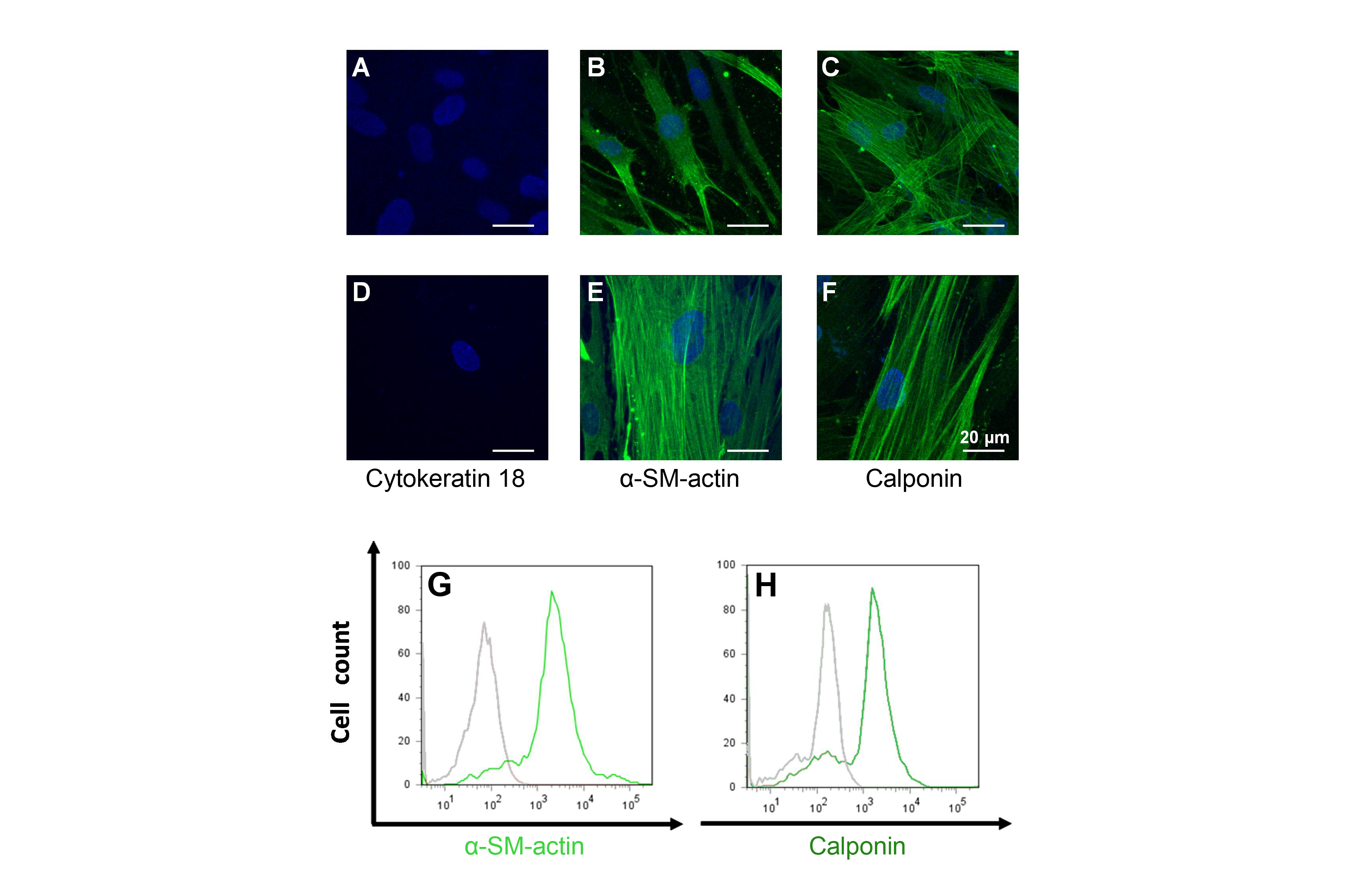

Supplement: Figure S1 — Bronchial smooth muscle cell phenotyping. Bronchial smooth muscle phenotype was assessed using both immunocytochemistry (A–F) and flow cytometry (G–H). Representative confocal microscopic images of cultured cells after 3-dimensional reconstruction of 20 sections of 0.25 microns thick (original magnification, ×600; scale bars = 20 µm). Cells were obtained from a control subject (A, B and C) or an asthmatic patient (D, E and F). Cells were stained with anti-cytokeratin 18 (A and D), anti-α-smooth muscle actin (α-SM-actin, B and E) or anti-calponin (C and F) primary antibodies, and by appropriate secondary antibodies (Alexa Fluor 488). Nuclei were stained in blue with DAPI. Representative flow cytometry histograms were obtained using bronchial smooth muscle cells from a control subject. α-smooth muscle-actin (G) or calponin (H) expression was assessed using irrelevant antibodies (gray lines) or specific antibodies (green lines). (TIF) [file pone.0086945.s001.tif]

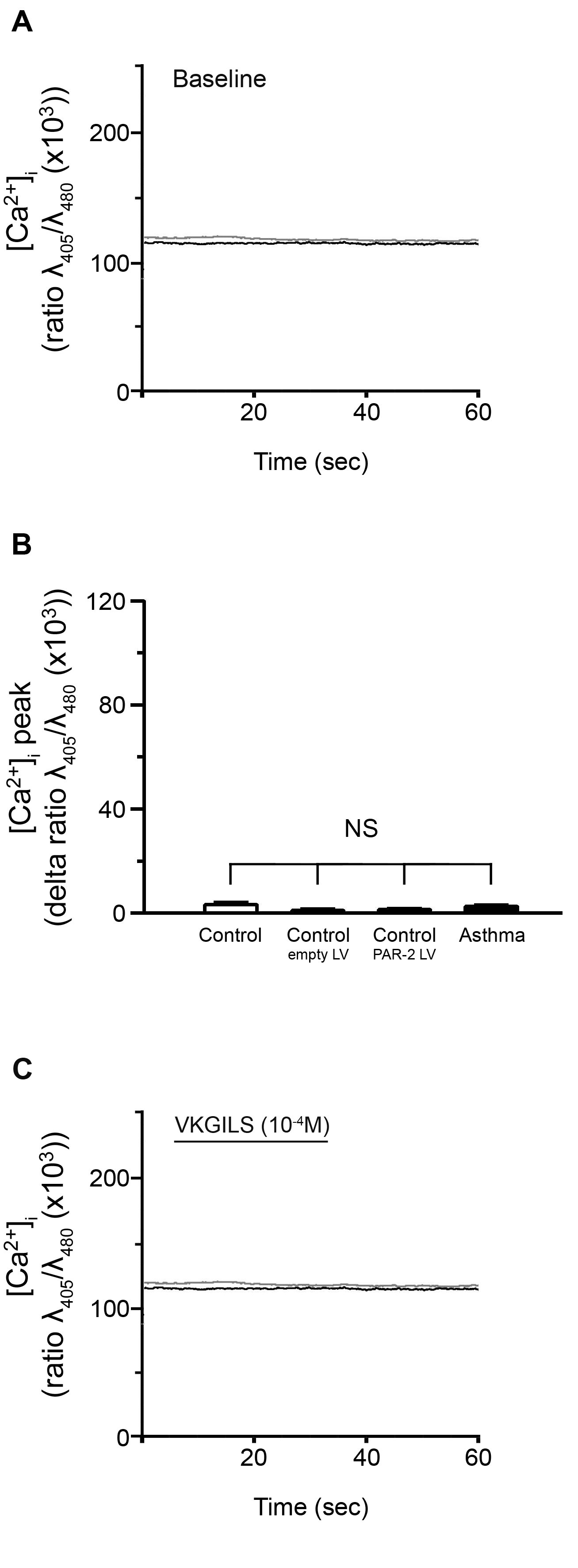

Supplement: Figure S2 — Calcium responses under baseline and VKGILS stimulations. Representative intracellular calcium baselines are presented in bronchial smooth muscle cells from asthmatic (black line) or control subject (grey line) (A). Relative calcium response ([Ca2+]i variations were assessed under baseline condition (B). The effects of lentivirus over-expressing PAR-2 (squared bars, n = 4) in control bronchial smooth muscle cells were evaluated as compared to both control bronchial smooth muscle cells transduced by control lentivirus (hatched bars, n = 4), control bronchial smooth muscle cells non transduced (white bars, n = 4) and asthmatic bronchial smooth muscle cells (black bars, n = 4). Results are expressed as mean ± SEM from a range of 12 to 19 cells per patient. Representative intracellular calcium responses following stimulation by 10−4 M VKGILS-NH2 for 30 sec are presented in bronchial smooth muscle cells from asthmatic (black line) or control subjects (grey line) (C). NS P non significant using Mann & Whitney test. (TIF) [file pone.0086945.s002.tif]

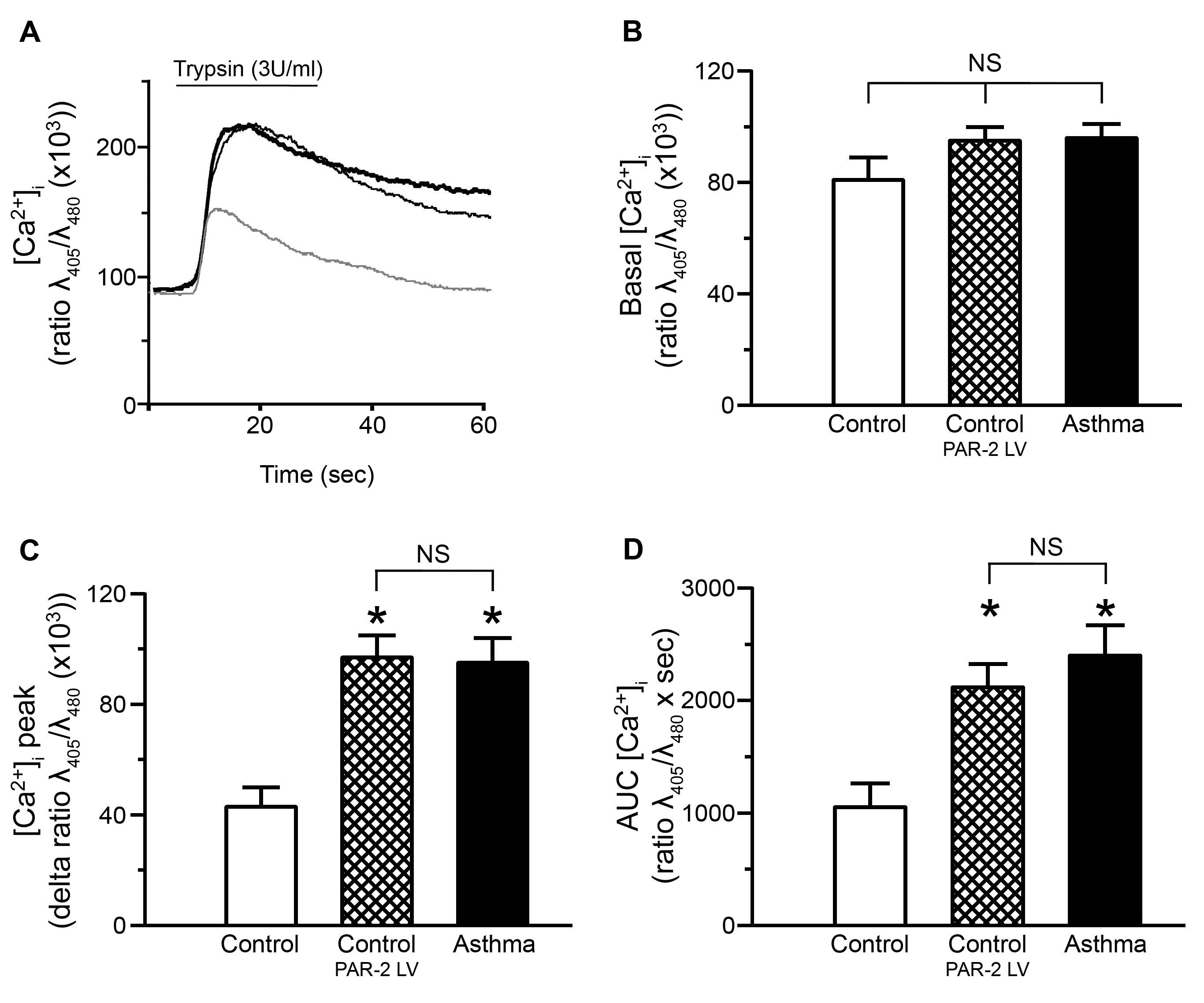

Supplement: Figure S3 — Increased trypsin-related calcium response in asthmatic bronchial smooth muscle cells. Representative intracellular calcium responses following stimulation by 3 U/ml trypsin for 30 sec are presented in asthmatic bronchial smooth muscle cells (bold black line), control bronchial smooth muscle cells (grey line) or control bronchial smooth muscle cells transduced with PAR-2 lentivirus (black line) (A). Basal calcium concentration (Basal [Ca2+]i, B), relative calcium response ([Ca2+]i peak, C) and area under the curve (AUC [Ca2+]i, D) were assessed from cell response to 3 U/ml trypsin. Non transduced bronchial smooth muscle cells were obtained from asthmatic (black bars, n = 4) and control subjects (white bars, n = 4). PAR-2 lentivirus-transduced bronchial smooth muscle cells were obtained from control subjects (squared bars, n = 4). Results are expressed as mean ± SEM from a range of 22 to 46 cells per patient. *P<0.05 using Mann & Whitney test. (TIF) [file pone.0086945.s003.tif]

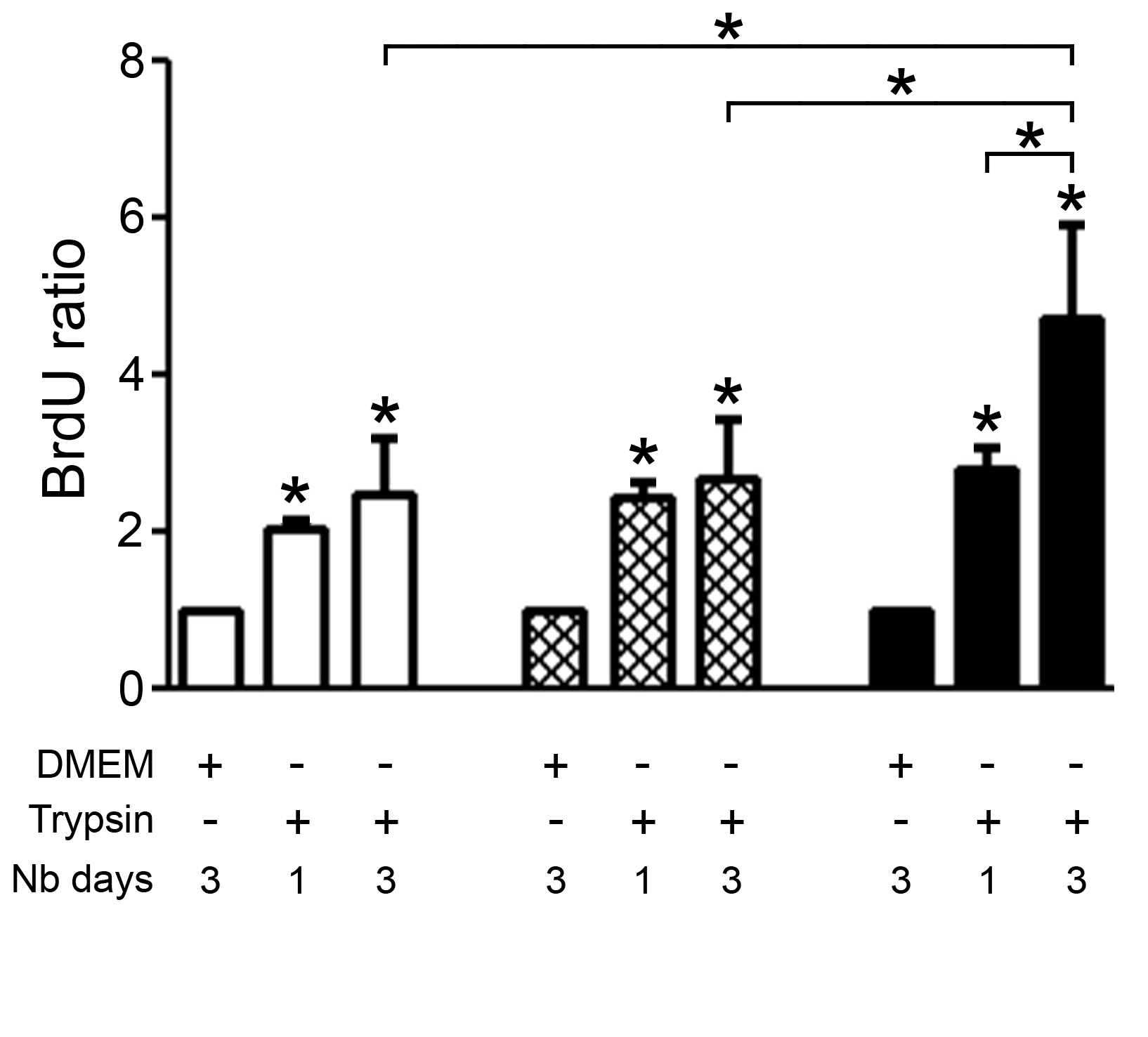

Supplement: Figure S4 — Increased asthmatic bronchial smooth muscle cell proliferation following repeated PAR-2 stimulations with trypsin. Proliferation was measured using BrdU incorporation following stimulation for 1 or 3 days by 30 mU/ml trypsin. Bronchial smooth muscle cells were obtained from asthmatic (black bars, n = 3) and control subjects (white bars, n = 3). Bronchial smooth muscle cells obtained from control subjects were also transduced with lentivirus over-expressing PAR-2 (squared bars, n = 3). Results are expressed as mean ± SEM. *P<0.05 using paired Wilcoxon-rank tests or Mann & Whitney tests. (TIF) [file pone.0086945.s004.tif]

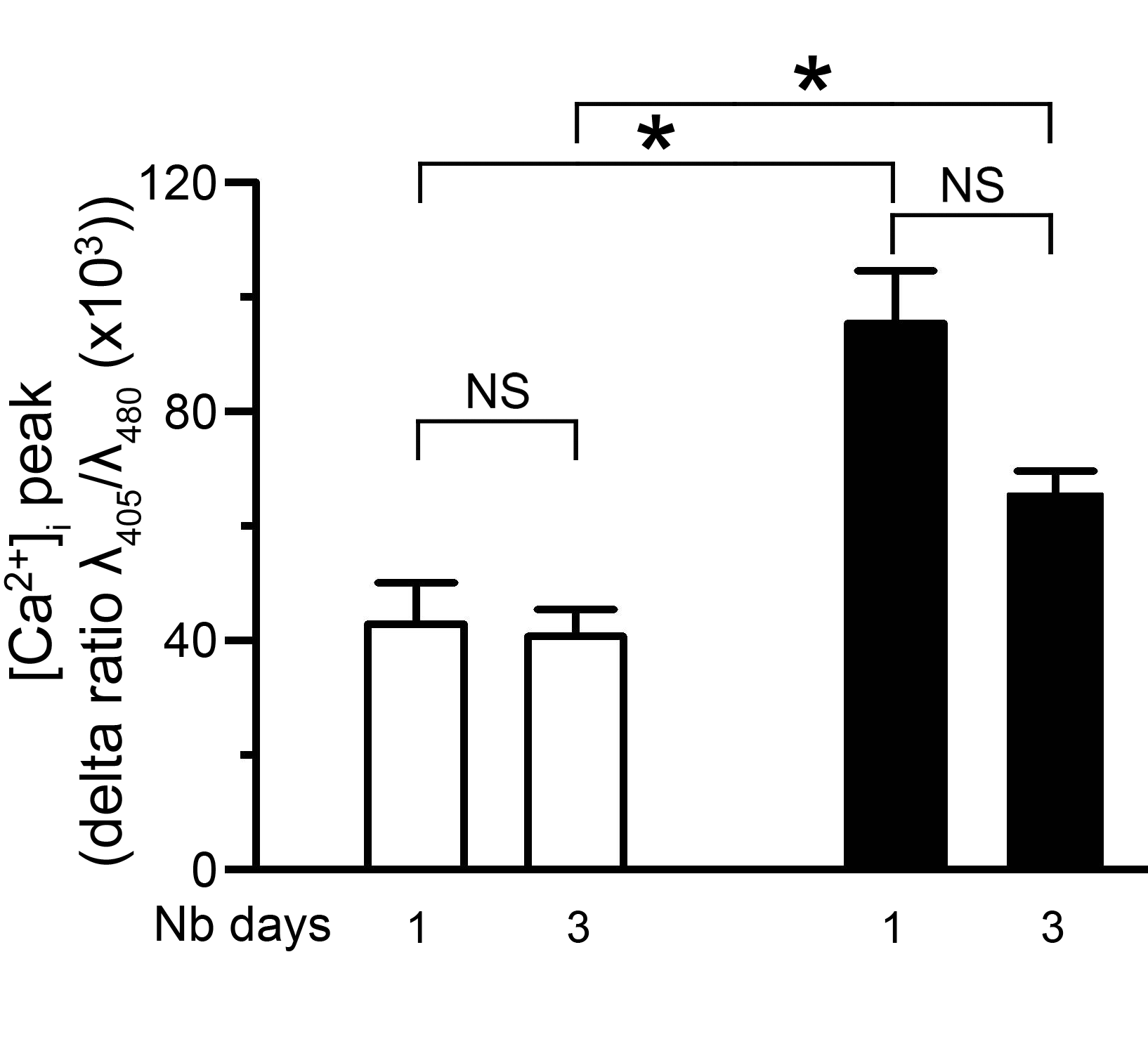

Supplement: Figure S5 — Increased trypsin-related calcium response in asthmatic bronchial smooth muscle cells under repeated stimulations. Relative calcium response ([Ca2+]i peak) were assessed by microspectrofluorimetry from the cell response to 3 U/ml trypsin after either 1 day stimulation or 3 days stimulation with 30 mU/ml trypsin. Bronchial smooth muscle cells were obtained from asthmatic (black bars, n = 4) and control subjects (white bars, n = 4). Results are expressed as mean ± SEM. Calcium responses were obtained from a range of 20 to 40 cells per patient. * P<0.05 using Mann & Whitney tests. NS non significant using paired Wilcoxon-rank tests. (TIF) [file pone.0086945.s005.tif]

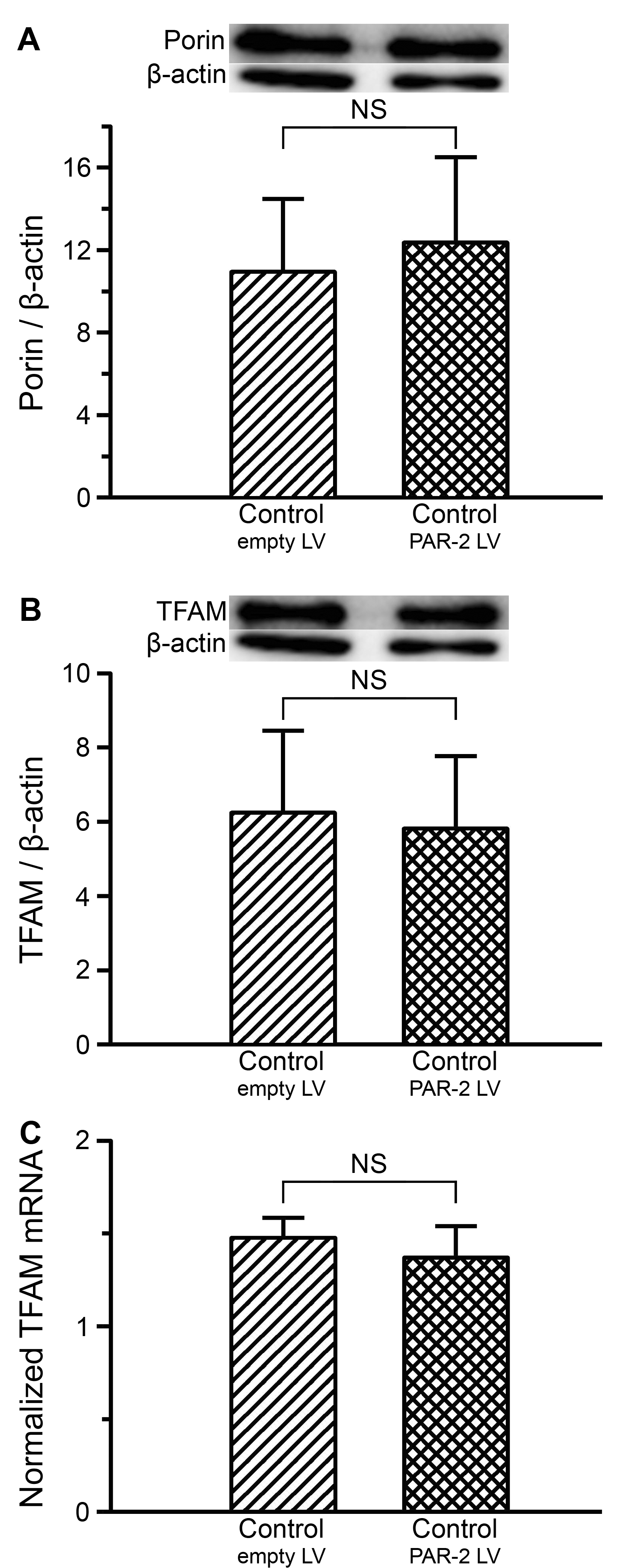

Supplement: Figure S6 — Unaltered mitochondrial biogenesis in control bronchial smooth muscle cells over-expressing PAR-2. The effects of lentivirus over expressing PAR-2 (squared bars) in control bronchial smooth muscle cells were compared to control bronchial smooth muscle cells transduced by control lentivirus (hatched bars). Porin level was assessed by western blot (A) and mitochondrial transcription factor A (TFAM) levels were assessed by both western blot (B) and quantitative RT-PCR (C). Representative blots stained with anti–porin, anti-TFAM or anti–β-actin antibodies are shown (A and B). Bronchial smooth muscle cells were obtained from control subjects (white bars, n = 4 for A, B, n = 6 for C). Results are expressed as mean ± SEM. *P<0.05 using paired Wilcoxon-rank tests. (TIF) [file pone.0086945.s006.tif]

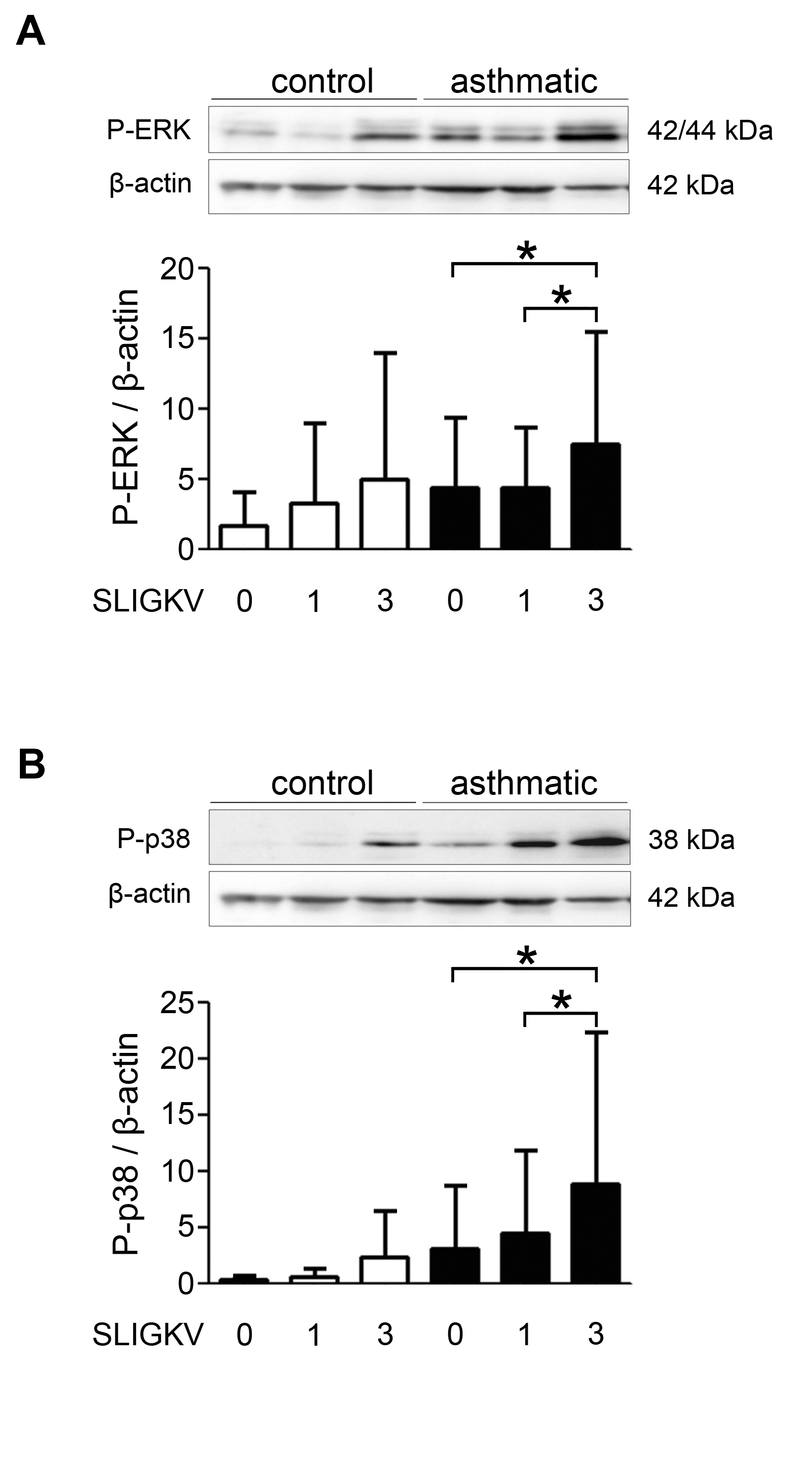

Supplement: Figure S7 — Increased asthmatic bronchial smooth muscle cell phosphorylation of ERK and p38 following repeated PAR-2 stimulations. Phosphorylation of ERK (A) and p38 (B) was measured using western blot following stimulation for 0, 1 or 3 days by 10−4 M SLIGKV-NH2. Representative blots stained with anti-Phospho-ERK (P-ERK), anti-Phospho-p38 (P-p38) and anti–β-actin antibodies are shown. Bronchial smooth muscle cells were obtained from asthmatic (black bars, n = 9) and control subjects (white bars, n = 5). Results are expressed as mean ± SD. *P<0.05 using paired Wilcoxon-rank tests. (TIF) [file pone.0086945.s007.tif]

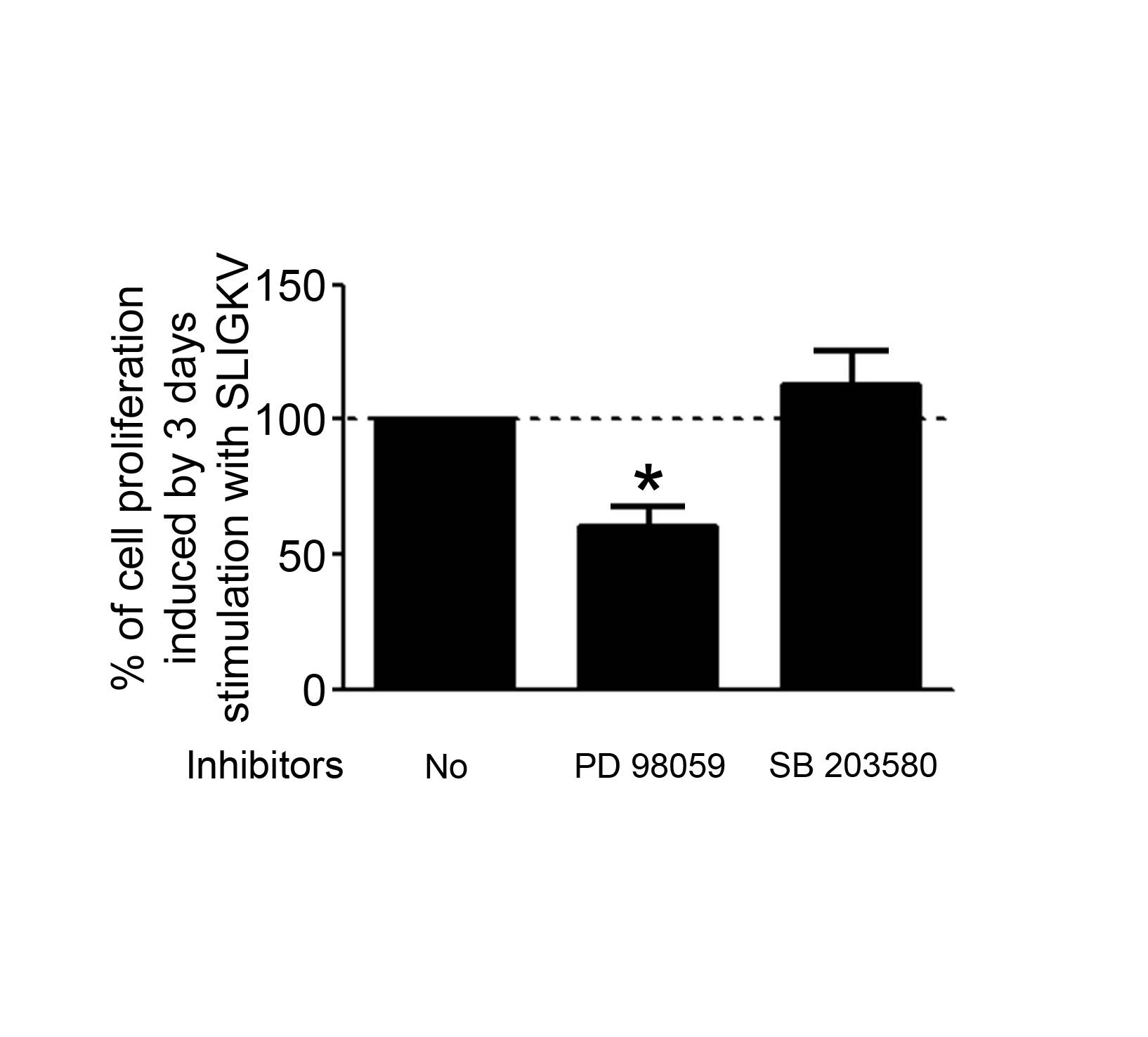

Supplement: Figure S8 — ERK inhibition decreased asthmatic bronchial smooth muscle cell proliferation following repeated PAR-2 stimulations. Cell proliferation was measured using BrdU incorporation following stimulation for 3 days by 10−4 M SLIGKV-NH2 in the absence or in the presence of ERK inhibitor (PD 98059) or p38 inhibitor (SB 203580). Bronchial smooth muscle cells were obtained from asthmatic patients (black bars, n = 4). Results are expressed as mean ± SEM of percentage of cell proliferation in the absence of inhibitor. *P<0.05 using paired Wilcoxon-rank tests. (TIF) [file pone.0086945.s008.tif]
