# Supplementary material for: Protease Activated Receptor-2 Expression and Function in Asthmatic Bronchial Smooth Muscle
Source: PLoS One. 2014 Feb 13;9(2):e86945. doi: 10.1371/journal.pone.0086945 (PMC3923726; doi:10.1371/journal.pone.0086945)
Supplement: Methods S1 — Supporting materials and methods. (DOC) [file pone.0086945.s009.doc]

**Supporting information**

**Protease activated receptor-2 expression and function in asthmatic bronchial smooth muscle**

Benoit Allard ^*,1,2^, Imane Bara ^*,1,2^, Guillaume Gilbert^1,2^, Gabrielle Carvalho^1,2^, Thomas Trian^1,2^, Annaig Ozier^1,2,3^, Jennifer Gillibert-Duplantier ^1,2^, Olga Ousova^1,2^, Elise Maurat^1,2^, Matthieu Thumerel^1,2,3^, Jean-François Quignard^1,2^, Pierre-Olivier Girodet^1,2,3^, Roger Marthan^1,2,3^, and Patrick Berger^1,2,3^

^*^ Authors contributed equally to this work

^1^Univ. Bordeaux, Centre de Recherche Cardio-thoracique de Bordeaux, U1045, Département de Pharmacologie, CIC 0005, F-33000 Bordeaux, France

^2^INSERM, Centre de Recherche Cardio-thoracique de Bordeaux, U1045, CIC 0005, F-33000 Bordeaux, France

^3^CHU de Bordeaux, Service d’Exploration Fonctionnelle Respiratoire, CIC 0005, F-33604 Pessac, France

Corresponding author: Pr. Patrick Berger, Centre de Recherche Cardio-thoracique de Bordeaux, INSERM, U1045, Université Bordeaux Segalen, 146 rue Léo Saignat. 33076 Bordeaux Cedex, France. Telephone: 33 5 57 57 16 94 Fax: 33 5 57 57 16 95

E-mail: [patrick.berger@u-bordeaux2.fr](mailto:patrick.berger@u-bordeaux2.fr)

**Supporting Materials and Methods**

**Ethics statement**

All patients gave their written informed consent to participate to the study, after the nature of the procedure had been fully explained. The study followed recommendations outlined in the Helsinki Declaration and received the approval from the local ethics committee (“CPP Sud-Ouest et Outre mer IV”).

**Study populations**

A total of 22 patients with mild to severe persistent asthma, and 33 non asthmatic subjects were prospectively recruited from the “Centre Hospitalier Universitaire (CHU)” of Bordeaux according to Global Initiative for Asthma criteria [1]. Bronchial specimens were obtained by either fiberoptic bronchoscopy or lobectomy, as described previously [2,3] (See Table 1 for patients’ characteristics). For non asthmatic subjects, we used bronchi from a grossly normal part of the lung specimen. The majority of non asthmatic subjects (32/33) presented distal lung carcinoma.

**Cell culture**

Human bronchial smooth muscle (BSM) cells were derived from bronchial specimens as previously described [2], from non asthmatic and asthmatic patients (See Table 1 for patients’ characteristics). Cells were cultured and maintained in DMEM (PAN Biotech, Brumath, France) containing 10% (v/v) FCS (PAN Biotech) and 4.5 g/L glucose, supplemented with 2 mM l-glutamine, 100 U/ml penicillin, 100 mg/ml streptomycin, and 0.25 mg/ml amphotericin B2 (antimycotic/antibiotic solution), 1 mM sodium pyruvate, and 1% (vol/vol) nonessential amino acid mixture (all from PAN Biotech), as previously described [4,5]. The medium was changed every 48–72 h.

We assessed cell purity by both immunocytochemistry and flow cytometry, on growth arrested cells using serum-free DMEM for 48 h (Figure S1). For immunocytochemistry, cells were rinsed in PBS and fixed with paraformaldehyde 4% for 10 min (PFA, Santa Cruz Biotechnology, Nanterre, France). Cells were rinsed in PBS and permeabilized with Triton X-100 0.5% (PAN Biotech). Cells were rinsed in PBS and nonspecific staining was blocked using PBS containing 4% Bovine Serum Albumin (BSA, Sigma-Aldrich, Saint-Quentin Fallavier, France) for 30 min. Monoclonal antibodies (Abs) diluted in PBS with 1% BSA, including anti–α-smooth muscle actin (α-SMA, Sigma-Aldrich), anti-calponin (Santa Cruz) and anti-cytokeratin 18 (Sigma-Aldricht) were incubated for 1 h. Control samples were treated similarly, using an unrelated Ab (mouse IgG; Sigma-Aldrich). After rinsing with PBS, the cells were incubated for 1 h with appropriate secondary Abs (Alexa Fluor 488 or 568) (Life Technologies, Saint Aubain, France). The nuclei are labeled with DAPI (Life Technologies). Slides were mounted with a drop of fluorescent mounting medium (Dako, Trappes, France) and observed under a fluorescence microscope (Nikon, Paris, France). For flow cytometry, BSM cells were harvested, fixed and permeabilized in 4% paraformaldehyde containing 0.1% saponin (Sigma-Aldrich). BSM cells were then labeled with primary antibodies indirectly labeled with appropriate fluorescent-conjugated secondary antibodies. Primary antibodies were either mouse monoclonal α-smooth muscle actin Ab or rabbit monoclonal anti-calponin Ab. Appropriate isotype control antibodies were systematically used. Analysis was performed using a double colour flow cytometry on a FACSCanto® (BD Biosciences, Pont de Claix, France). Cell purity was stable between passages 2 and 5, and was always more than 80% using flow cytometry and more than 90% using immunocytochemistry (Figure S1) in both asthmatic and non asthmatic BSM cells.

All experiments were performed on phenotypically confirmed BSM cells between passages 2 and 5 [2]. In each experiment, we compared BSM cells of the same passages from both non asthmatic and asthmatic subjects. Depending on the experiments, BSM cells were seeded on 96-well plates for BrdU incorporation, on 6-well plates for immunoblotting and flow cytometry, on 12-well plates for lentiviral transduction and quantitative RT-PCR.

**BSM cell stimulation**

A synthetic peptide with a sequence corresponding to the tethered ligand domain of PAR-2 (SLIGKV in humans) has been employed experimentally to activate PAR-2 without proteolytic cleavage [6]. We also used the reverse peptide VKGILS-NH_2_ as negative control for SLIGKV-NH_2_ experiments [4]. Both SLIGKV-NH_2_ and VKGILS-NH_2_ (PolyPeptide Group, Strasbourg, France) were used on growth arrested BSM cells at 10^-4^ M and, were changed every 24 h for 1 to 3 days for proliferation, PAR-2 expression, transduction cell and calcium study. Similarly, trypsin was used to activate PAR-2 at 30 mU/ml and was changed every 24 h for 1 to 3 days for proliferation assay and at 3 U/ml for calcium study. In order to determine the EC50 to SLIGKV-NH_2_, cell proliferation was assessed using SLIGKV-NH_2_ at various concentrations from 10^-9^ M to 10^-4^ M.

**PAR-2 expression**

PAR-2 expression was evaluated by flow cytometry, western blot and quantitative RT-PCR.

For flow cytometry, BSM cells were fixed with paraformaldehyde 4% (Santa Cruz Biotechnology) for 15 min in ice and washed twice with 0.5% BSA (Sigma-Aldrich). Mouse IgG anti-human PAR-2 (SAM11; Santa Cruz Biotechnology) or isotype control (Sigma-Aldrich) at the same concentration of 20 μg/ml was added to BSM for 30 min in ice. After an additional wash, secondary FITC-conjugated goat anti-mouse IgG (Immunotech, Marseille, France) was then added for another 30 min in ice. Cells were analyzed for their fluorescence intensity using FACSCanto® (BD Biosciences). Normalized median fluorescence intensities were calculated by dividing PAR-2 median fluorescence intensity by isotype control median fluorescence intensity. Results were presented as mean normalized median fluorescence intensity ± SEM.

For western blot, BSM cells were rinsed in cold PBS, and immunoblotting was performed on cell protein extracts [7]. Cells were lysed with 50 mM Tris pH 7.4, 150 mM NaCl, 1% Triton X-100, 1% Igepal, 2 mM EDTA, supplemented with complete protease inhibitors (Roche, Meylan, France). Lysates were cleared by a centrifugation at 12,000 g at 4°C, and protein concentration determined using Lowry (Bio-Rad Laboratories, Marnes-la-Coquette, France). Samples were resolved on 5–20% SDS-PAGE gels and transferred to nitrocellulose membranes (Amersham Biosciences, Fontenay-sous-Bois, France). The immunoblots were then developed using 1 μg/ml of mouse monoclonal anti-human PAR-2 antibody (SAM11), 0.8 μg/ml of mouse anti-human β-actin, or irrelevant antibody (all from Santa Cruz Biotechnology). A biotinylated swine secondary antibody (Dako) and a streptavidin–biotinylated horseradish peroxidase complex (Dako) were used for amplification [5]. Immunoblots were revealed by enhanced chemiluminescence Immobilon (Millipore, Molsheim, France). Blot images were acquired using BioCaptMW (Thermo Fisher Scientific, Illkirch, France), and band densities were quantified using ImageJ® software (National Institutes of Health).

For quantitative RT-PCR, BSM cell total RNA was extracted and reverse transcribed into cDNA using AMV reverse transcriptase (Sigma-Aldrich) as previously described [2]. Briefly, real time PCR was performed on a Rotorgene 2000 (Corbett Research, Mortlake, Australia), using specific PAR-2 primers previously optimized [3] (NM_005242) forward 5’-CCGAACTAAGAAGAAGCACC-3’ reverse 5’-AGAAAAAGCCAATAAGCACAT-3’; or other primers specific for tyrosin 3-monooxygenase/tryptophan 5’-monooxygenase activation protein, zeta polypeptide (YWHAZ) (NM_003406), forward 5’-GGGGATGTGGAATTTTTATACAAG-3’ and reverse 5’-CGCCAGGACAAACCAGTATGTAG-3’; for hypoxanthine phosphoribosyltransferase 1 (HPRT-1) (NM_000194), forward 5’-GTGAAAAGGACCCCACGAA-3’ and reverse 5’-AGTCAAGGGCATATCCTACAACAA-3’; and for human acidic ribosomal phosphoproteins (PO) (NM_001002), forward 5’-CAACGGGTACAAACGAGTC-3’ and reverse 5’-CTTCCTTGGCTTCAACCTTAG-3’.

The efficiency of the PCR reactions was always >90%. The specificity of the amplified PCR products was examined with the melting curve analysis, and also in 2% agarose gel containing SYBER® Green (Invitrogen, Life Technologies, Saint Aubin, France). The RT-PCR expression of PAR-2 was then presented as an arbitrary unit and normalized to endogenous references (geometric averaging of three internal control genes; *i.e.* YWHAZ, HPRT-1, and PO) according to geNorm software [8].

**Lentivirus over-expressing PAR-2**

The genomic library clone IRATp970H0715D ([http://www.imagenes-bio.com](http://www.imagenes-bio.com/)), containing PAR-2 ORF cDNA was used to amplify the coding sequence of the gene using the following primers (forward 5’- ATGCGGAGCCCCAGCGCG-3’ and reverse 5’-TCAATAGGAGGTCTTAAC-3’). The obtained PCR fragment was then cloned into a transfer lentiviral plasmid containing a GFP reporter gene (Plate-forme de vectorologie SFR TransBioMed, Univ Bordeaux, France). Three plasmids were then co-transfected in human embryonic kidney cells (293T): (i) transfer plasmid vector containing PAR-2 sequence, (ii) packaging plasmid pVSV-G (Trono, Geneva, Switzerland) and (iii) the HIV structure plasmid (Trono) to produce replication-deficient lentiviral particles. Control lentiviral particles were also produced similarly using non modified transfer plasmid. Infectious titers were determined by transducing the 293T cell with serial dilution of viral supernatant. GFP expression was quantified 5 days later by flow cytometry [9].

BSM cells (400 000 cells/ml) were then transduced with optimized concentration of either control or PAR-2 lentiviral particles in 12-well plates. The efficacy of transduction was assessed using both flow cytometry and real time quantitative RT-PCR seven days post transduction.

**Microspectrofluorimetric measurement of cytosolic calcium**

The Ca^2+^-sensitive fluorescent probe indo-1 was used to record changes in intracellular calcium concentration ([Ca^2+^]_i_) in cells, as described previously [5]. BSM cells were incubated during 30 minutes in a Kreb’s modified solution containing 5 µM of indo-1 penta-acetoxymethyl ester (indo1/AM; I-1223, Molecular Probes, Life Technologies) at room temperature. Cells were then washed 15 minutes in indo-1/AM free solution and maintained at room temperature in the same solution. The indo-1 calcium probe was excited at 355 nm and the emitted fluorescence was recorded at 405 nm and 480 nm. The fluorescence ratio (λ_405_/λ_480_) representing the [Ca^2+^]_i_ was calculated and recorded using the pCLAMP software. After 5 seconds, each cell was stimulated during 30 seconds with SLIGKV-NH_2_ or VKGILS-NH_2_ peptide at 10^-4^ M. It should be noted that, for calcium analysis following repeated PAR-2 stimulation by SLIGKV-NH_2_, a wash out of SLIGKV-NH_2_ was performed during Indo-1 loading and washing. For analysis, the calcium peak induced by the peptide stimulation and the area under curve were determined using the Clampfit 10.2 software (Axon Instrument, California, United States).

**Cell proliferation**

Cell proliferation was evaluated using BrdU incorporation, as described previously [3]. BSM cells were growth-arrested using serum deprived culture medium for 24 h and stimulated with either the SLIGKV-NH_2_ or VKGILS-NH_2_ peptide, at the concentration of 10^-4^ M, for 1 to 3 days. DNA synthesis was assessed using the BrdU kit according to the manufacturer’s instructions (Roche Diagnostics, Meylan, France). Briefly, BSM cells were incubated with 10 μM BrdU for 24 h at 37°C. Cells were fixed and denaturized for 30 min at room temperature and incubated with the anti-BrdU Ab for 2 h at room temperature. Absorbance was measured at 370 nm in a microplate reader (SPECTROstarNano, BMG Labtech, Champigny sur Marne, France). All experiments were performed in triplicate. Results were expressed as a normalized ratio of BrdU incorporation, *i.e.* absorbance for test condition divided by absorbance for control serum-deprived condition.

**Cell transduction**

AKT, ERK and p38 phosphorylation were analyzed following 1 to 3 days challenge with SLIGKV-NH_2_, using western blot as previously described [3]. Briefly, the immunoblots were developed using phospho-specific antibodies against AKT, ERK or p38 (Cell Signaling Technology, Saint Quentin Yvelines, France) and, after stripping, anti-total AKT, ERK or p38 (Cell Signaling Technology) and anti-human β-actin (Sigma-Aldrich) for 14 h at 4°C. As for PAR-2, we used the same methods described for amplification, revelation, images acquisition and analysis.

Cell proliferation following 3 days of stimulation with SLIGKV-NH_2_ was also evaluated in the absence or in the presence of ERK inhibitor PD98059 (Calbiochem, up to 50 µM) or p38 inhibitor SB203580 (Calbiochem, up to 10 µM), as previously described [3].

Mitochondrial mass was assessed by the porin content using western blot, and mitochondrial biogenesis was assessed by peroxysome proliferator-activated receptor  γ co-activator-1α (PGC-1α), nuclear respiratory factor-1 (NRF-1) and mitochondrial transcription factor A (TFAM) contents using both quantitative RT-PCR and western blot, as previously described [2]. Briefly for western blot, the immunoblots were developed using mouse anti-human porin (MitoSciences, Mundolsheim, France), mouse anti-human β-actin (Sigma-Aldrich), mouse anti-human PGC-1α, anti-human NRF-1, or anti-TFAM (all purchased from Abcam, Paris, France). For quantitative RT-PCR, primers were as follows: for TFAM (NM_003201), forward 5‘-GAAGTCGACTGCGCTCCC-3’ and reverse 5’-ACTCCGCCCTATAAGCATC-3’; for PGC-1α (NM_013261), forward 5’-GTCACCACCCAAATCCTTATT-3’ and reverse 5’-GGCGATCTTGAACGTGAT-3’; for NRF-1 (NM_005011), forward 5’-AAGATCAGCAAACGCAAACAC-3’ and reverse 5’-CCCGTACCAACCTGGATAAGT-3’.

**Statistical analysis**

The statistical analysis was performed with NCSS software (NCSS 1997®; NCSS Statistical software, Kaysville, Utah). Values are presented as the mean ± SD or SEM. Statistical significance was analyzed by paired Wilcoxon tests and Mann and Whitney tests. A p value < 0.05 was considered statistically significant.

**References**
